# Regular pulse rate but irregular heart rate?

**DOI:** 10.1007/s12471-016-0831-9

**Published:** 2016-04-04

**Authors:** B. Bellmann, C. Gemein, P. Schauerte

**Affiliations:** 1 Department of Cardiology, Charité Berlin Campus Benjamin Franklin, Berlin, Germany; 2 Department of Cardiology, University Hospital Gießen, Gießen, Germany; 3 Kardiologie an der Rudower Chaussee, Berlin, Germany; 4 Department of Cardiology, University Hospital, Technical University Aachen RWTH, Aachen, Germany

This 30-year-old male patient attended the emergency department because of dizziness accompanied by self-assessed regular pulse rates of 30–40 bpm. Physical examination was normal but cardiac auscultation revealed frequent premature beats. A subsequent 12-lead ECG documented the arrhythmia (Fig. 1). Similar episodes had occurred 12 months ago but thorough cardiac evaluation, including echocardiography and exercise stress testing, did not show any signs of underlying organic heart disease. Thus, empiric beta-blockade with metoprolol 95 mg 1‑0‑1 was initiated but did not relieve the symptoms. Instead, beta-blockade yielded fatigue due to arterial hypotension and was discontinued.

**Fig. 1:**
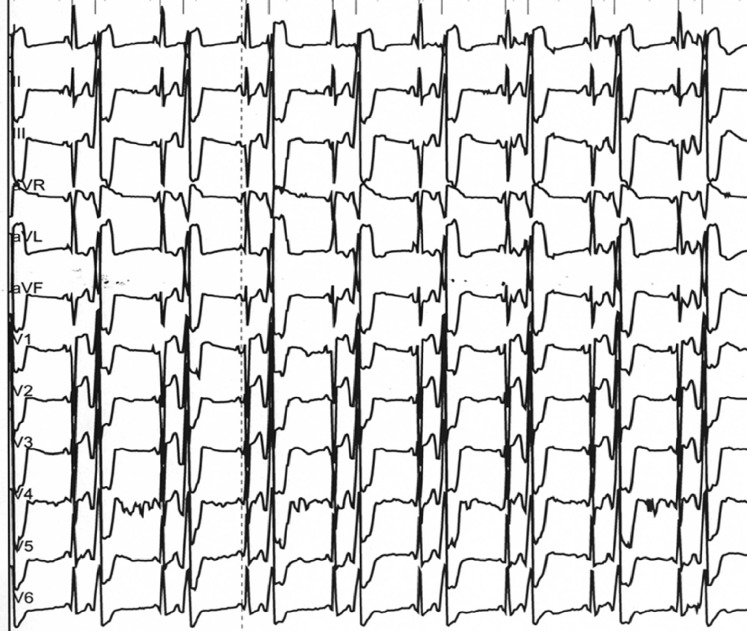
Resting ECG of the patient

What is the diagnosis of the arrhythmia, which can be made just by the morphology of the premature beats in the 12-lead ECG? Why did the patient report slow and regular pulse rates? What is the prognosis of the arrhythmia? And finally, is there any cure for this arrhythmia?

## Answer

You will find the answer elsewhere in this issue.

